# Editorial: Is Prosocial Behavior Always Good for the Workplace? On the Direction and Strength of the Relationship Between Prosocial Behaviors and Workplace Outcomes

**DOI:** 10.3389/fpsyg.2020.01886

**Published:** 2020-08-19

**Authors:** Abira Reizer, Bella L. Galperin, Meni Koslowsky

**Affiliations:** ^1^Department of Behavioral Sciences, Ariel University, Ariel, Israel; ^2^John H. Sykes College of Business, The University of Tampa, Tampa, FL, United States

**Keywords:** OCB, workplace, prosocial behavior, positive, negative

## Introduction

In an increasingly dynamic, competitive and global environment, organizations in the twenty-first-century are required to accommodate to rapid changes (Bolino et al., 2018). In these uncertain times, organizations can profit from prosocial activities in a variety of ways, including improved service quality and the enhanced ability to attract and retain job seekers. The literature has also shown that employees can benefit from prosocial behaviors and organizational citizenship behaviors (OCBs) (e.g., improved performance appraisals, higher status, greater social ties, and job promotions; for recent reviews, see Podsakoff et al., 2009; Bolino and Grant, 2016; Bolino et al., 2018).

Much of the existing research on prosocial behaviors has focused primarily on the positive outcomes overlooking the negative effects. More recently, organizational researchers have examined the dark side of prosocial behavior. Prosocial acts can also have several personal costs such as: increased burnout and role overload, work family conflicts, and even reduced productivity (Bolino et al., 2018). Employees may feel obligated to help and sacrifice their own personal resources. Based on the depletion perspective which claims that personal resources are limited, prosocial behaviors may consume additional cognitive, emotional, and physical resources with undesirable consequences (Bolino and Grant, 2016; Bolino et al., 2018).

This special issue provides a balanced perspective by exploring the benefits and costs of prosocial behaviors in the workplace and addressing some of the conceptual gaps in the literature. Taken together, the seven papers in this special issue advances the field in three ways. First, the studies contribute to the ongoing debate on the beneficial and/or harmful outcomes of prosocial behavior. Specifically, three studies focus on the positive aspects of OCB and prosocial behavior, one study emphasizes the negative consequences, and remaining three studies presents prosocial behavior as a double-edged sword by examining both positive and negative aspects. Second, we respond to Organ's (2018) call for more diverse methodologies and approaches in his recent review of OCB. In this special issue, there are four quantitative papers using different methods (e.g., multi-source data and daily reports of OCB), one qualitative study, and two papers take a purely theoretical approach. Finally, this special issue contributes to our understanding of the mechanisms that explain the bright and dark sides of prosocial behaviors, as well as the personal and contextual factors that may impact the direction and strength of the relationship between prosocial behaviors and workplace outcomes.

## Overview of the Articles in This Research Topic

In this special issue, three studies examine the benefits of prosocial behavior. The paper by Freidlin and Littman-Ovadia discusses the central role of character strengths from a theoretical perspective in promoting desirable prosocial behaviors in the workplace. Since the character strengths framework is rarely used in the literature, this novel approach can provide insight into the mechanisms of prosocial behavior.

Similarly, Wang et al. also investigate the positive outcomes of prosocial behavior by examining a moderated mediation model that links ethical leadership to subordinate taking charge behavior. Using a sample of leader–subordinate dyads in China, their results show that subordinates' social exchange mediates the relationship between ethical leadership and taking charge as an outcome.

Yaakobi and Weisberg further expand on the benefits of prosocial behavior by examining the mechanisms that facilitate or inhibit prosocial behaviors within organizations. The findings of their two studies revealed that occupational efficacy emerged as an antecedent of OCB in predicting performance. Moreover, employees' and managers' beliefs in the employee's work team efficacy moderated the relationship between OCB and different facets of performance (quality, creativity, and efficiency).

The negative impact of OCBs is addressed in the qualitative study conducted by Lavee and Pindek. The authors focus on delineating the informal resources that are invested as part of OCBs directed toward customers (OCBCs), as well as the costs associated for employees. This study contributes to better understanding OCBCs and the growing trend to “do more with less in the workplace.”

Contrary to the former studies, Shukla and Kark provide a theoretical framework which examines both positive and negative impacts of prosocial behavior. The authors introduce pro-social behavior as an antecedent of creative deviance and develop a multi-level model of the moderators of this relationship. The model suggests that prosocial motivation can increase creative deviance, which ultimately increases both positive (e.g., creative performance and innovation) and negative (e.g., waste of resources) outcomes.

Lavy paper also contributes to a more balanced examination of prosocial behavior. The paper examines the daily dynamics of prosocial behaviors among a group of teachers and focuses on the interplay of daily perceived supervisor and colleague support, OCB, and daily positive and negative emotional experiences. The study presents new findings supporting the dual role of social support and emotions as both antecedents and outcomes of OCB.

Finally, Reizer et al.'s paper explores the general mechanisms and moderators that explain the bright and dark sides of prosocial behaviors. Overall, findings from their two studies suggest that performing OCB can enhance work-family facilitation (WFF), with the effect being stronger for workers with low avoidance levels. However, OCB can be harmful in terms of work family facilitation among individuals who are higher on attachment avoidance.

## Conclusion

This special issue offers a more comprehensive perspective of prosocial behavior by providing both the positive and negative sides of the phenomenon. Given the increased importance of prosocial research in today's volatile environment, the current set of papers answers critical questions on direction and strength of the relationship between prosocial behaviors and workplace outcomes. We hope that the current set of papers will also inspire others to further explore these issues and contribute in developing a more integrative model of prosocial behavior in organizations.

## Author Contributions

All authors listed have made a substantial, direct and intellectual contribution to the work, and approved it for publication.

## Conflict of Interest

The authors declare that the research was conducted in the absence of any commercial or financial relationships that could be construed as a potential conflict of interest.
